# Tract-Based Spatial Statistics: Application to Mild Cognitive Impairment

**DOI:** 10.1155/2014/713079

**Published:** 2014-05-12

**Authors:** Yau-Yau Wai, Wen-Chuin Hsu, Hon-Chung Fung, Jiann-Der Lee, Hsiao-Lung Chan, Ming-Lun Tsai, Yu-Chun Lin, Yih-Ru Wu, Leslie Ying, Jiun-Jie Wang

**Affiliations:** ^1^Department of Medical Imaging and Radiological Sciences, Chang Gung University, Taoyuan County, Taiwan; ^2^Department of Radiology and Intervention, Chang Gung Memorial Hospital, 5 FuHsing Street, Linkou, Taoyuan County, Taiwan; ^3^Department of Neurology, Chang Gung Memorial Hospital, Chang-Gung University College of Medicine, Linkou, New Taipei 333, Taiwan; ^4^Dementia Center, Chang Gung Memorial Hospital, 5 FuHsing Street, Linkou, Taoyuan County, Taiwan; ^5^Department of Electrical Engineering, Chang Gung University, 259 WenHua 1st Road, Taoyuan County, Taoyuan, Taiwan; ^6^Department of Biomedical Engineering, University at Buffalo, The State University of New York, Buffalo, NY 14260, USA; ^7^Department of Electrical Engineering, University at Buffalo, The State University of New York, Buffalo, NY 14260, USA; ^8^Neuroscience Research Center, Chang Gung Memorial Hospital, 5 FuHsing Street, Linkou, Taoyuan County, Taiwan; ^9^Institute of Radiological Research, Chang Gung University, Chang Gung Memorial Hospital, 5 FuHsing Street, Linkou, Taoyuan County, Taiwan; ^10^Healthy Aging Research Center, Chang Gung University, 259 WenHua 1st Road, Taoyuan County, Taoyuan, Taiwan

## Abstract

*Rationale and Objectives*. The primary objective of the current investigation was to characterize white matter integrity in different subtypes of mild cognitive impairment (MCI) using tract-based spatial statistics of diffusion tensor imaging. *Materials and Methods*. The study participants were divided into 4 groups of 30 subjects each as follows: cognitively healthy controls, amnestic MCI, dysexecutive MCI, and Alzheimer's disease (AD). All subjects underwent a comprehensive neuropsychological assessment, apolipoprotein E genotyping, and 3-tesla MRI. The diffusion tensor was reconstructed and then analyzed using tract-based spatial statistics. The changes in brain white matter tracts were also examined according to the apolipoprotein E **ε**4 status. *Results*. Compared with controls, amnestic MCI patients showed significant differences in the cerebral white matter, where changes were consistently detectable in the frontal and parietal lobes. We found a moderate impact of the apolipoprotein E **ε**4 status on the extent of white matter disruption in the amnestic MCI group. Patients with AD exhibited similar but more extensive alterations, while no significant changes were observed in dysexecutive MCI patients. *Conclusion*. The results from this study indicate that amnestic MCI is the most likely precursor to AD as both conditions share significant white matter damage. By contrast, dysexecutive MCI seems to be characterized by a distinct pathogenesis.

## 1. Introduction

Dementia refers to a clinical syndrome of acquired intellectual disturbances produced by brain dysfunction [1]. Mild cognitive impairment (MCI) is considered to be the clinical transition stage between normal aging and dementia [2]. Evidence suggests that subjects with MCI tend to progress to probable Alzheimer's disease (AD) at a rate of approximately 10% to 15% per year [2]. However, MCI is a clinically heterogeneous syndrome, with some patients showing isolated memory impairments (i.e., amnestic MCI) and others with isolated executive function impairments (i.e., dysexecutive MCI). Importantly, Yaffe et al. [3] demonstrated that dysexecutive MCI patients are less likely to convert to dementia but have higher 5-year mortality rates than amnestic MCI.

MRI studies of dementia have been mainly focused on the assessment of hippocampal and entorhinal atrophy [4–6]. Global brain atrophy on conventional MRI has been proposed as a marker for advanced MCI [7, 8], but the low specificity limits its clinical value [9, 10]. Similarly, neuropsychological tests can be considered as screening tools for MCI but are not adequate for diagnosis. Diffusion tensor imaging (DTI) is MRI technique that can noninvasively measure macroscopic axonal organization in the central nervous system [11, 12]. Because of the potential for investigating white matter integrity and fiber connectivity* in vivo*, this technique has been widely applied to study brain disorders [13, 14].

Three eigenvalues and three corresponding eigenvectors can be derived from the diffusion tensor. The largest eigenvalue is often referred to as the axial diffusivity (longitudinal diffusivity (LD), in the study, in order not to be confused with Alzheimer's disease) and the average of the second and third eigenvalues as radial diffusivity (RD) [15]. In white matter, diffusion tensor is usually modeled as cylindrically symmetric [16, 17]. The increase of the mean diffusivity (MD) can be related to an increase in either LD or RD. The reduction of FA can be related to either a decrease of diffusion in the longitudinal direction (AD) or an increase in the transverse direction (RD). Many studies attributed the changes in RD as related axonal injury or demyelination process [18].

Conventional DTI data are often analyzed in a region of interest approach. Because the directional information is encoded by the signal intensity, great care is required for all imaging processing procedures (e.g., registration and normalization). Tract-based spatial statistics (TBSS) [19, 20] is a new whole brain voxelwise analysis method that provides more reliable alignment of the white matter tracts and less bias from smoothing than conventional voxel-based methods. The data-driven approach of TBSS is particularly useful in a disorder like MCI, for which regional patterns of brain abnormalities are not fully determined. Thus, the aim of the present study was to investigate white matter changes in two MCI subgroups, dysexecutive MCI and amnestic MCI, using a TBSS approach. Imaging patterns were also compared with those of AD patients and cognitively healthy controls.

## 2. Materials and Methods

The study was approved by our institutional review board and complied with the tenets of the Declaration of Helsinki. Each participant or participant's legal guardian gave written informed consent for participation.

### 2.1. Participants and Clinical Workup

The study participants (*n* = 120) were divided into 4 clinical groups of 30 subjects each as follows: cognitively healthy controls, amnestic MCI, dysexecutive MCI, and AD. The clinical workup included a thorough medical history, physical examination, and neuropsychological testing. Four key cognitive domains were assessed: memory, executive function, language, and visuospatial skills. All subjects were screened for the presence of depressive symptoms using either the Hamilton depression rating scale (healthy control) or the Cornell scale for depression in dementia (AD, amnestic MCI, and dysexecutive MCI). Patients with depression were excluded.

The diagnosis of AD was made using the NINCDS-ADRDA criteria [21]. The clinical dementia rating was used to quantify the severity of symptoms of dementia. MCI patients were diagnosed after an extensive clinical evaluation. The clinical phenotype of MCI was determined according to the criteria by Petersen [1]. The amnestic MCI group exhibited an isolated memory impairment without deficits in other cognitive domains. Patients with dysexecutive MCI had a relatively focal dysfunction in the executive domain, while memory, language, and visuospatial skills remained within the normal range. Cognitively healthy controls were free of cognitive impairment as judged by clinical assessment, neuropsychological testing, and clinical dementia rating.

### 2.2. Apolipoprotein E Genotyping

Genomic DNA was extracted from leukocytes in samples of whole blood, following a standard salting-out technique. Apolipoprotein E genotypes were detected by polymerase chain reaction followed by restriction fragment length polymorphism analysis.

### 2.3. Image Acquisition

Images were acquired using a 3-tesla MR scanner (Magnetom Trio with TIM, Siemens, Erlangen, Germany). T2WI FLAIR and T1WI magnetization-prepared rapid acquisition gradient echo images were acquired to rule out concomitant neurological disorders. DTI was acquired using a spin-echo EPI sequence with the following parameters: TR/TE/flip angle = 7500 ms/83 ms/90°, field of view = 256 mm^2^, and matrix size = 128 × 128. Diffusion-weighting gradients were applied in 64 noncollinear directions with a diffusion weighting of 1000 s/mm^2^. Sixty-four contiguous axial slices were obtained to cover the majority of the brain with an isotropic voxel size of 2 mm. The single average acquisition time was 8 minutes 47 seconds.

### 2.4. Image Processing and Analysis

Diffusion analysis was performed and the white matter skeleton was identified using TBSS, part of FSL (http://fsl.fmrib.ox.ac.uk/fsl/fsl4.0/tbss/index). In brief, the reconstructed fractional anisotropy [16, 17] of each individual was initially aligned to the MNI template. The parameters from the nonlinear registration were then applied to the diffusion indices, including MD/LD/RD [16, 17]. The spatial normalization was achieved by 12-parameter affine transformation. Both followed the recommended procedures and parameters by TBSS. The aligned diffusion indices were then projected onto a fiber tract-derived skeleton and fed into the voxelwise cross-subject statistics. Data from each of the diffusion indices were compared by the Randomise 2.1 software [22, 23]. Multiple comparison correction was done by the familywise error correction with a threshold of *P* < 0.05. We identified abnormal white matter tracts based on the atlas prepared at Johns Hopkins University [24]. The correlation between neuropsychiatry measures and the diffusion indices was assessed using the regression model in the SPM (Wellcome Trust Centre for Neuroimaging, UK). The effect of the apolipoprotein E *ε*4 allele was determined by dividing participants in either apolipoprotein E *ε*4 (*ε*2/*ε*4, *ε*3/*ε*4, and *ε*4/*ε*4) or non-*ε*4 (*ε*2/*ε*2, *ε*2/*ε*3, and *ε*3/*ε*3) carriers. Cognitive performances were compared between carriers and noncarriers using Student's *t*-test. The TBSS approach was also applied to compare the imaging patterns according to the apolipoprotein E *ε*4 carrier status.

## 3. Results

**Figure 1:**
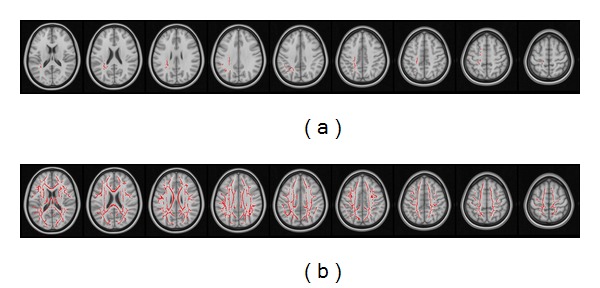
Fractional anisotropy in the white matter regions of the study participants. Compared with normal controls, fractional anisotropy is reduced within the white matter skeleton of both patients with amnestic MCI (a) and AD (b). The left of the figure corresponds to the subject's right side.

Table 1 shows the general characteristics of the study participants. The neuropsychological tests are reported in Table 2. The results from TBSS analysis did not identify significant white matter changes in dysexecutive MCI patients compared with cognitively healthy controls.  Figure 1 shows the changes in fractional anisotropy along the white matter skeleton of patients with amnestic MCI (a) and AD (b) compared with healthy controls. Both groups of patients exhibited a widespread fractional anisotropy decrease within the white matter tracts. Changes in the amnestic MCI group were restricted to the frontal and parietal lobes, while AD patients consistently exhibited more extensive damage which was suggestive of a progressive disease process.

**Figure 2:**
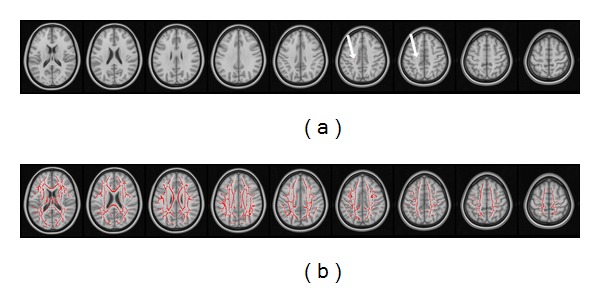
Radial diffusivity in the white matter regions of the study participants. Compared with normal controls, radial diffusivity is increased within the white matter skeleton of both patients with amnestic MCI (a) and AD (b). The pattern of such changes is consistent with the observed reductions in fractional anisotropy. The arrows indicate the locations of the fiber tracts showing significant changes.

Figure 2 shows an increase in RD along the white matter skeleton of both amnestic MCI (a) and AD (b) patients compared with healthy controls. The location was consistent but significantly smaller than the corresponding areas identified in Figure 1. The arrows indicate the location of the most significant areas of increased RD. These results suggest that an increased RD was presumably driving a reduction of fractional anisotropy in both AD and amnestic MCI patients.

**Figure 3:**
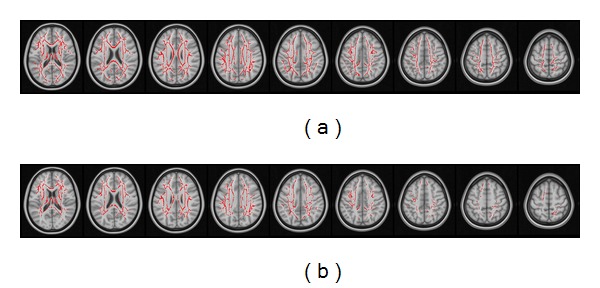
MD and longitudinal diffusivity in the white matter regions in patients with AD. Compared with normal controls, MD (a) and longitudinal diffusivity (b) are significantly increased within the white matter skeleton of patients with AD. The observed increases are largely consistent with the corresponding reductions in fractional anisotropy.

Figure 3 shows the corresponding increases of MD (a) and LD (b) in AD patients. No significant changes of these parameters were found in the amnestic MCI group. The observed increase of MD in AD patients was largely in accordance with the corresponding fractional anisotropy changes (depicted in Figure 1(b)). The pattern of LD increases was similarly consistent, with the only exception being the absence of significant changes in the occipital lobe.

**Figure 4:**
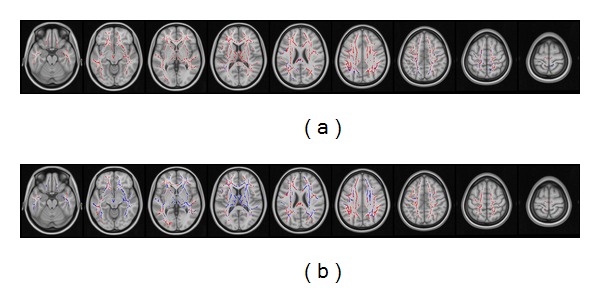
Effect of the apolipoprotein E *ε*4 status on white matter changes in amnestic MCI patients. The diffusion indices are examined in amnestic MCI patients after stratification according to apolipoprotein E *ε*4 status. In (a), the decrease of fractional anisotropy is marked in blue, while the increase in MD is reported in red. In (b), the increases of LD and RD are marked in blue and red, respectively.

The effect of the apolipoprotein E *ε*4 allele on white matter changes in amnestic MCI patients is shown in Figure 4. In (a), the decrease of fractional anisotropy is marked in blue, while the increase in MD is reported in red. In (b), the increases of LD and RD are marked in blue and red, respectively. Compared with healthy subjects, amnestic MCI patients without the apolipoprotein E *ε*4 allele exhibited larger and more striking changes in both fractional anisotropy and RD. Similarly, more extensive areas of MD and LD changes were evident in apolipoprotein E *ε*4 noncarriers.

In all study groups, we found no correlation between changes in diffusion indices and neuropsychiatric measurements, even after allowance for age and/or education levels.

## 4. Discussion

There are three main findings in this study. First, we have shown that AD and amnestic MCI may share a common neurodegenerative substrate which impairs white matter integrity. The results from this study indicate that this process can be efficiently visualized in a noninvasive manner using DTI-TBSS. Second, AD patients showed more extensive white matter damage compared with amnestic MCI subjects, supporting the view that amnestic MCI is the most likely precursor to AD. Third, the apolipoprotein E *ε*4 carrier status affected the extent of white matter involvement in amnestic MCI patients. Importantly, we found no evidence of significant white matter damage in dysexecutive MCI, suggesting highly distinct pathways of neurodegeneration compared with amnestic MCI. Moreover, there was no correlation between diffusion indices and neuropsychological test scores, suggesting no direct association between DTI-TBSS findings and MCI.

### 4.1. White Matter Changes in AD and MCI

Diffusion tensor imaging is sensitive to white matter damage and has been previously used to examine entire neural networks and their integrity in AD and MCI [25, 26]. The observed changes in diffusion indices have been mainly attributed to alterations in the white matter structure. Compared with normal controls, both amnestic MCI and AD are characterized by increases in RD and MD and a reduction in fractional anisotropy. The increase in RD has generally been ascribed to alterations in myelin sheaths, which can be related to the observed changes in both MD and fractional anisotropy. As these indices are altered in amnestic MCI and AD, it is feasible that both conditions are characterized by an altered brain myelination.

The results from this study indicate a significant overlap between the affected regions of amnestic MCI and AD patients. The changes in amnestic MCI patients were chiefly located in the frontal and parietal regions. However, the extension of damage was significantly greater in AD patients, suggesting that amnestic MCI is the most likely precursor to AD.

LD did not differ in both amnestic and dysexecutive MCI, while AD patients showed an increase of both LD and RD. In normal aging, an increased LD reflects a reduced axonal packing in white matter structures due to thinning of myelin or decrease in axonal diameter. This phenomenon in turn results in an increased diffusivity in all orientations within a voxel [25, 27]. Using MRS, Meyrhoff et al. provided suggestive evidence of diffuse axonal injury and membrane alterations in AD patients [28]. Our results indicate that LD changes seem to be restricted to AD patients.

We found no significant association between neuropsychological testing and the results of DTI-TBSS analysis. However, these results should be interpreted cautiously as cognitive performances were assessed only at the study admission; additionally, all neuropsychological scores were distributed in a narrow range. In any case, it is noteworthy that white matter changes on DTI-TBSS do not necessarily reflect changes in cortical functions.

In this study, there were no significant alterations in diffusion indices of dysexecutive MCI patients, suggesting that changes in white matter structure do not play a major role in this condition. Alternatively, such alterations could be so subtle that they escaped detection by means of DTI-TBSS. Further studies are necessary to shed more light on the neuroradiological alterations specific to dysexecutive MCI.

### 4.2. Impact of APOE  *ε*4 Carrier Status on White Matter Changes

The apolipoprotein E *ε*4 allele is the most widely recognized genetic risk factor for sporadic AD [29] and affects, even for nondemented elders, the levels of cognitive performance [30]. In the present study, amnestic MCI patients who carried at least one apolipoprotein E *ε*4 allele showed a nonsignificant trend toward lower scores on the executive function and memory measures compared with those without. In contrast, we found no impact of the apolipoprotein E *ε*4 status on both visuospatial and language processing.

The results from the DTI-TBSS analysis demonstrated that the apolipoprotein E *ε*4 status had a moderate impact on the extent of white matter disruption in amnestic MCI patients. In subjects without the apolipoprotein E *ε*4 allele, we found that RD and LD were significantly increased, which can be related to the observation of an increased MD and a reduced fractional anisotropy. It is noteworthy that the impact of the apolipoprotein E *ε*4 status on cognitive functions may vary in different ethnic groups [31, 32]. Interestingly, the apolipoprotein E *ε*4 allele is less prevalent in people of Chinese ancestry [33]. This variability has been shown to influence the association of this polymorphism with different phenotypes [33, 34]. For this reason, the observed association between the apolipoprotein E *ε*4 status and the extent of white matter disruption in our amnestic MCI patients should be confirmed in multiethnic populations. In addition, future studies should assess how the presence of white matter abnormalities reflects associative cortical functioning in MCI patients.

### 4.3. Comparison with Previous Studies

Bosch et al. [35] have previously identified a general neural network which seems to be specifically disrupted in AD. However, the authors failed to identify specific white matter changes in amnestic MCI patients compared with healthy controls. In contrast, Zhuang et al. [36] reported a significant fractional anisotropy reduction in the frontal, temporal, and parietal lobes of amnestic MCI patients. The fractional anisotropy changes identified in our study are similar to those reported by Zhuang et al. [36], albeit being located in a smaller region. This discrepancy can be attributed to the different sample sizes used in the studies. It is also important to note that MCI is not a diagnostic entity as AD is; consequently, a number of studies use the term MCI but do not use Petersen's criteria [1]. Between-study heterogeneity in MCI severity may also explain the discrepancy. In accordance with Zhuang et al. [36], our study identified significant signal changes of the white matter in amnestic MCI patients. This result deserves further independent scrutiny.

### 4.4. Study Limitations

The findings of our study should be interpreted within the context of the following limitations. First, a well-established model for tensor signal processing in the gray matter is still lacking. For this reason, we limited our DTI-TBSS analysis to the white matter skeleton. The analysis of gray matter diffusion properties may be challenging, but this knowledge may ultimately help define the correlation between white matter changes and cortical functioning in amnestic MCI and AD patients. Furthermore, the observed changes in LD/RD, as detected in the white matter skeleton, can be controversial noticeably in the region where the fiber distribution is complicated or where the fibers cross [37, 38]. Therefore, the interpretation of the observed white matter change should be exercised with great care.

Second, in this study we did not correlate* in vivo* imaging findings with postmortem analysis of brain tissue. Furthermore, given the cross-sectional design, we cannot infer causality between white matter changes and the conversion of amnestic MCI to AD. Notwithstanding these limitations, the results from this study indicate that amnestic MCI is the most likely precursor to AD as both conditions share significant white matter damage. By contrast, dysexecutive MCI seems to be characterized by a distinct pathogenetic mechanism.

## 5. Conclusion

Amnestic MCI is the most likely precursor to Alzheimer's disease because both conditions share significant white matter damage as assessed by tract-based spatial statistics. Dysexecutive MCI seems to be characterized by a distinct pathogenesis.

## Figures and Tables

**Table 1:** General characteristics of the study participants.

	Controls	Dysexecutive	Amnestic	AD
Number of subjects	30	30	30	30
Age (years)	67.2 ± 5.6	65.9 ± 7.6	68.6 ± 8.3	73.9 ± 9.4
Range	61–80	50–84	56–84	54–83
Median	65.5	65	69	76.5
Sex (males/females)	18/12	12/18	17/13	12/18
Education (years)	10.1 ± 4.7	8.0 ± 3.5	8.3 ± 5.3	7.3 ± 6.4
Clinical dementia rating score	0.00 ± 0.00	0.12 ± 0.22	0.47 ± 0.13	1.00 ± 0.57
Apolipoprotein E status				
*ε*4 carriers	2	4	11	5
*ε*4 noncarriers	28	23	16	23
Unknown	0	3	3	2

Data are given as means ± standard deviation or counts, as appropriate.

**Table 2:** Neuropsychological test scores.

	Controls	Apolipoprotein E *ε*4 noncarriers	Amnestic MCI apolipoprotein E *ε*4 carriers	Sum	Dysexecutive MCI	AD
Mean global score						
Mini-mental status examination	28.23 (1.30)	25.25 (3.87)	25.09 (4.18)	25.10 (3.87)	27.63 (1.47)	15.07 (6.34)
Mean memory score						
Word sequence learning-recall	3.17 (1.93)	0.47 (0.64)	0.55 (0.93)	0.45 (0.74)	2.00 (1.68)	0.04 (0.20)
Logic memory II	11.60 (2.72)	8.25 (3.66)	8.18 (4.21)	7.97 (3.72)	9.87 (2.42)	3.15 (1.87)
Mean executive function score						
Semantic association of verbal fluency	33.40 (6.82)	31.81 (4.04)*	25.73 (7.16)*	29.57 (6.03)	32.83 (7.37)	14.55 (6.20)
Wisconsin card sorting test Completed Categories	5.20 (1.13)	3.67 (1.91)	3.82 (1.89)	3.69 (1.77)	2.60 (1.35)	1.88 (2.76)
Mean visuospatial score						
3D block construction models	28.6 (0.77)	27.31 (2.60)	26.91 (4.11)	27.27 (3.1)	28.03 (1.65)	18.41 (10.50)
Mean language score						
Object naming test	16.00 (0.00)	15.94 (0.25)	16.00 (0.00)	15.97 (0.18)	15.97 (0.18)	13.13 (3.65)

Data are given as means (standard deviation); *Significant differences at *P* < 0.05 (*P* = 0.0225).
